# Research in Perioperative Care of the Cancer Patient: Opportunities and Challenges

**DOI:** 10.3390/curroncol30010091

**Published:** 2023-01-15

**Authors:** Juan P. Cata

**Affiliations:** 1 Department of Anesthesiology and Perioperative Medicine, The University of Texas MD Anderson Cancer Center, Houston, TX 77030, USA; jcata@mdanderson.org; 2 Anesthesiology and Surgical Oncology Research Group, Houston, TX 77030, USA

**Keywords:** cancer, surgery, anesthesia, research, challenges

## Abstract

The theory that the perioperative period is critical for oncological outcomes has been a matter of extensive preclinical and clinical research. Basic science research strongly supports the notion that surgical stress, anesthetics, and analgesics influence the mechanisms of cancer progression. Hence, it is hypothesized that perioperative interventions that impact mechanisms or predictors of tumor progression can also affect patients’ survival. As a result of that hypothesis, clinical researchers have conducted many retrospective studies. However, much fewer randomized controlled trials have been performed to investigate whether surgery itself (minimally invasive versus open procedures), anesthetics (volatile anesthetics versus propofol-based anesthesia), analgesics (opioids versus opioid-free anesthesia), and blood transfusions (transfusions versus no transfusions) modify the survival of patients with cancer. Unfortunately, randomized controlled trials have failed to translate the preclinical results into clinical outcomes. In this review, I will highlight the challenges of translating basic science to clinical outcomes. We will also point out opportunities for future research.

## 1. Introduction

This year, millions of patients are facing a diagnosis of cancer worldwide [1]. Unfortunately, millions of new cancer cases will be diagnosed by 2030 due to the aging population—the baby boomer generation [2]. Coincidentally, the United Nations has mandated a substantial reduction in all premature deaths by 2030, including cancer-related mortality. Therefore, a significant effort will be made by healthcare systems in developed and developing countries to reduce cancer-associated deaths significantly. Considering this, and despite advances in new therapies to cure cancer—most notably immunotherapies—surgery remains a cornerstone therapy for the ten most prevalent cancers when timely detected.

For a long time, surgeons and scientists have noted that the perioperative period is critical for the long-term success of cancer-related surgical procedures. Exaggerated inflammation and high levels of catecholamines, immunosuppression, a proangiogenesis state, and the release of circulating tumor cells into the bloodstream during and after surgery were theorized as the mechanisms involved in cancer progression [3]. Elegant in vitro experiments and studies in rodents supported the theory [4,5]. Consequently, several lines of investigations were initiated to discover molecular targets or mechanisms that would serve as regulators of mechanisms for cancer progression associated with surgical stress and inflammation.

One of the proposed mechanisms was the modulation of the stress response from anesthetics [6]. Hence, during the early years of this century, anesthesiologists launched multiple clinical studies to understand whether anesthetics or analgesics could contribute to cancer recurrence or progression months to years after surgery. Early retrospective studies suggested a protective effect of regional anesthesia over intravenous-based opioid analgesia on cancer recurrence [7,8]. However, as researchers conducted and reported the results of well-designed randomized controlled studies, the excitement or concerns about anesthetics’ potential beneficial or harmful effects on cancer recurrence started to fade. Thus, the gap between preclinical observations suggesting a negative impact of certain anesthetics or analgesics and their null effect on human cancer outcomes became clear from those studies. Contrarily, a growing body of evidence from preclinical and human studies indicated that two classes of drugs—nonsteroidal anti-inflammatory drugs (NSAIDs) and antagonists of the beta-adrenergic receptors (beta-blockers)—could significantly influence the trajectory of oncological outcomes after cancer surgery [9].

This review will discuss and summarize the literature on the advantages, limitations, and challenges of conducting research in the perioperative period of patients with cancer.

### 1.1. Challenges with In Vitro Studies

**Figure 1:**
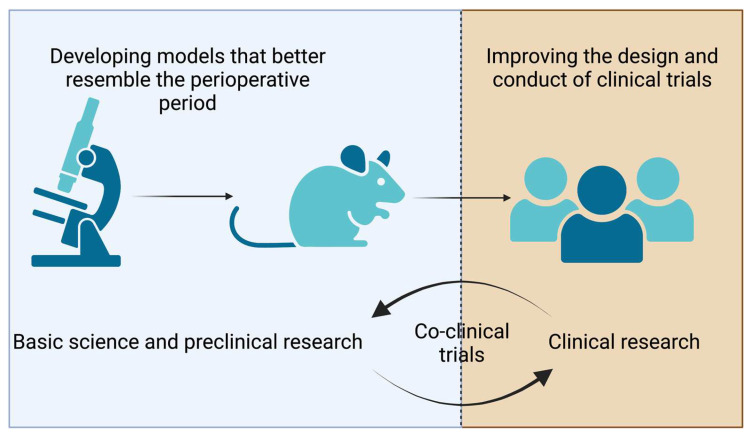
The figure illustrates opportunities in preclinical and clinical research. Co-clinical trials consist of laboratory investigations and clinical trials conducted simultaneously, thus enabling real-time data integration to better understand and predict patients’ responses to treatments.

Clinical research relies on preclinical investigations and vice versa (Figure 1). Preclinical science is key to understanding cellular mechanisms involved in tumorigenesis and cancer progression. One of the goals of in vitro studies is to dissect how cellular signaling affects cell behaviors participating in tumor growth (proliferation and clone formation assays) and metastasis (cell migration and invasion assays). Investigators have used these in vitro assays to evaluate how anesthetics (volatile or propofol) or analgesics such as opioids, local anesthetics, and NSAIDs modulate the proliferation, clone formation, and metastasic properties of malignant cell lines from different origins.

To understand some of the challenges presented by such studies on anesthetics and cancer recurrence, let us take two recent studies as examples. In 2022, Wu et al. reported the effects of propofol on the metabolism of colorectal cancer [10]. Propofol is the most common intravenous anesthetic administered for total intravenous anesthesia. In Wu’s work, the investigators used two different cell lines (HCT-16 and LoVo) [10] and treated them with “low” and “high” dosages of propofol in a time-dependent manner. As reported in many other investigations using a similar approach, when HCT-16 and LoVo were incubated with a high dose of propofol for 48–72 h, they demonstrated a lower metastatic capacity than the controls; a phenomenon that was attributed to a significant reduction in glycolysis [10]. The readers of that work could be impressed by the findings in that paper. However, let us examine a critical issue with the experimental design [10]. In humans undergoing surgery, prolonged infusions of propofol—in days—are extremely rare, except for those that may require intensive care unit admission. Hence, I could argue that the experimental approach in Wu’s work significantly deviated from clinical care.

Patients with glioblastoma have a dismal prognosis, and surgery is still considered part of the standard of care for resectable tumors. Volatile anesthetics such as sevoflurane are routinely used in patients undergoing craniotomies for glioma resections. Therefore, let us consider a publication examining the effect of sevoflurane—a commonly used volatile anesthetic—on glioma cells. Zhu et al. theorized that the circ_0037655/miR-130a-5p/RPN2 axis would be implicated as a critical mechanism linked to the anticancer effect of sevoflurane in glioma. To test the effect of sevoflurane on glioma cells’ behavior, the investigators used T98G and LN229 cells and three different in vitro experimental tests: proliferation, clonogenic, and invasion assays. For each assay, cells were incubated with increasing concentrations (1.5%, 3%, and 4.5%) of sevoflurane for 6 h in a 5% CO_2_ incubator at 37 °C. In these experimental conditions, sevoflurane—in a dose-related manner—caused cell death, inhibited colony formation, and reduced cell invasion. Important in vitro results! However, one could argue that the clinical relevance of these apparently startling results is questionable since the experimental conditions do not resemble clinical practice. Briefly, concentrations of 3% and 4.5% of sevoflurane during six hours are rarely administered during craniotomies for glioma resections. In support of this argument, we should consider another study by Lai et al. in which glioma cells were treated for 4 h with concentrations ranging between 1% and 4% of sevoflurane. Interestingly, clinically relevant concentrations of sevoflurane 1–2% did not influence cell behaviors, while higher concentrations (3–4%) stimulated colony formation, migration, and invasion [11].

Taken together, these and multiple other in vitro studies, including ours, we can clearly identify a challenge—modeling in vitro assays resembling perioperative conditions. We should use short-term exposure and clinically relevant concentrations of the studied anesthetics to overcome such difficulties. Fortunately, most investigations demonstrate that low in vitro concentrations of anesthetics given for a short period have small or negligible effects on cancer cell behaviors.

To add complexity, the vast majority of the in vitro studies investigating the effect of anesthetics and analgesics on mechanisms of cancer progression also ignore a major component of cancer—its microenvironment. Anesthetics and analgesics also modulate cellular elements of the tumor microenvironment, such as macrophages, lymphocytes, and endothelial cells. For instance, our group has demonstrated that local anesthetics, including lidocaine, stimulate the killing activity of natural killer cells—a key cellular component of the innate immune response against cancer [12,13]. However, these studies were conducted in very controlled in vitro conditions that ignore other immune modulators of the immune system known to regulate the function of natural killer cells, such as T regs or tumor-associated macrophages.

From our and others’ investigations, the challenge is to create experimental conditions that would consider the effect of anesthetic drugs on the tumor microenvironment.

### 1.2. In Vivo Preclinical Studies

Animals have been essential in cancer and anesthesia research. Animals, especially rodents, are typically used to study the activity of anticancer therapies in vivo, including surgery. A laparotomy model in rats with an intact immune system has been used to investigate the impact of anesthetics on natural killer cells [14]. Studies using this model demonstrate that the most volatile anesthetics, compared to propofol, cause a certain degree—mild to moderate—of immunosuppression when given during rodent surgery [15]. Remarkably, NSAIDs and stimulants of the immune system can mitigate the negative impact of surgery and volatile anesthetics on the immune system [16,17].

One of the limitations of using rats in oncological research is, in fact, the presence of a competent immune system. For this reason, immunodeficient mice are most frequently used to facilitate the growth of human cell lines and to evaluate the impact of anticancer drugs. Thus, most investigators have used immunodeficient mice to investigate how anesthetics, analgesics, and other drugs used in the perioperative period impact tumor growth and cancer progression in vivo. As we pointed out, with in vitro studies, several methodological aspects should be considered when interpreting the results from in vivo investigations. For this, we will return to Zhu’s work on the effect of sevoflurane on glioma cells. The investigators anesthetized mice with 4.5% sevoflurane after inoculating them subcutaneously with T98G cells [18]. Unanesthetized mice were used as controls. Five weeks after inoculation, the tumors were excised and measured [18]. Zhu reported that sevoflurane had significantly reduced tumor volume, which was counteracted when cells were transfected with circ_0037655 [18]. In stark contrast, when we examined Lai’s work, short exposure to sevoflurane (4%) twice after u87 glioma cell inoculation significantly inhibited tumor growth.

Major differences between Zhu’s and Lai’s investigations highlight challenges with in vivo studies [11,18]. First, investigators typically use different cancer cell lines; Zhu used T89G cells and Lai experimented with U87 cells [11,18]. Second, the duration of exposure to different drugs—in this case, sevoflurane—is also a source of heterogeneity among different investigations. Third, and perhaps most notorious, is the location of tumor growth. In Zhu’s work, cells were implanted subcutaneously, providing a different microenvironment to that found in the brain, as in the case of Lai’s work [11,18].

Another recurrent methodological issue encountered when examining the impact of volatile and intravenous anesthetics is their repetitive administration to animals. Let us examine as an example the work by Liu et al., who investigated the effect of lidocaine on ovarian cancer [19]. The authors reasoned that lidocaine—the only local anesthetic given systemically—would inhibit the metastatic potential of ovarian cancer in using a syngeneic mouse model. For this, mouse ID8 ovarian cancer cells were implanted intraperitoneally (i.p.), followed by daily infusions, during three consecutive days, of lidocaine or saline using the same route of administration (i.p.). A week later, the animals were treated again with lidocaine, cisplatin, their combination, or saline. The combination of lidocaine and cisplatin showed a statistically significant reduction in tumor growth compared to lidocaine, cisplatin, and controls (saline), which supported the authors’ in vitro work [19]. It is worth highlighting the fact that Liu used a syngeneic model with accepted dosages of experimental drugs [19]. The combination of lidocaine and cisplatin showed a statistically significant reduction in tumor growth compared to lidocaine, cisplatin, and controls (saline), which supported the authors’ in vitro work [19]. However, the manner of lidocaine and cisplatin administration is unconventional to the current clinical care, which limits the research findings [19].

Let us consider another example. Using an orthotopic mouse model of breast cancer, Freeman et al. investigated the perioperative effect of lidocaine and cisplatin [20]. The authors used a murine model in which 4T1 breast cancer cells were inoculated in the inguinal mammary gland. In the study, Freeman administered an intravenous lidocaine bolus of 1.5 mg/kg followed by a 2 mg/kg/h infusion in combination with a dose of intravenous cisplatin, 3 mg/kg, during tumor resection. The authors reported a significantly lower pulmonary colony count in mice treated with lidocaine plus cisplatin than in cisplatin alone or the controls [20]. It is worth noting that in humans, there are no reports of the co-administration of cisplatin and lidocaine during breast cancer surgery, thus limiting the clinical application (translation) of the findings [20].

Taken together, in vitro and in vivo studies are critical to advance the understanding of how perioperative therapies could influence mechanisms of cancer progression. However, equally important is that such studies are conducted based on experimental designs that resemble the perioperative period.

## 2. Challenges with Studies in Humans

Traditionally, evidence-based medicine categorizes clinical studies according to grades or strength of evidence. Randomized controlled trials rank high in the level of evidence. By contrast, retrospective studies are weak in the level of strength. In 2006, Exadaktylos et al. published an important retrospective study suggesting a negative association between the use of intravenous opioid analgesia during breast cancer surgery and cancer recurrence [7]. The importance of that study was not in the findings *per se*, which were categorically revoked in a randomized controlled trial [21], but in laying the ground for investigating the association between different anesthesia and analgesia techniques and cancer recurrence.

Many retrospective studies have been published since the publication of Exadaktylos’ study. While retrospective studies are a relatively quick and inexpensive method for establishing associations, it is worth remembering that they are inundated with methodological issues (Table 1). The most important limitation of retrospective studies is that they cannot establish a cause-and-effect relationship between the intervention—in our case, anesthesia or analgesia technique—and one or more outcomes, such as recurrence-free or overall survival. In retrospective studies, confounding, defined as the difference in the risk of the outcome—say, cancer recurrence—between one anesthesia technique over another, can be explained entirely or partly by imbalances in known or unknown factors, also referred to as measured, unmeasured but measurable, or unmeasurable confounders.

Let us take Exadaktylos’ work to exemplify some challenges with retrospective studies and highlight the issue of confounding. In the study, Exadaktylos reported that the proportions of women in the paravertebral and general anesthesia groups who received adjuvant therapies (i.e., chemotherapy and radiation) were not statistically significant. Furthermore, the authors did not comment, for instance, on the time-to-initiation of the adjuvant treatments, which is a known independent predictor of cancer-specific survival [22]. Therefore, in Exadaktylos’ study, the lack of adjusting for the time-to-initiation of adjuvant therapies could explain why women in the general anesthesia group had more recurrences than in the paravertebral group [22].

There are strategies to mitigate confounding in retrospective studies (i.e., matching, propensity scores, external adjustment, or instrumental variable analysis). Let us consider, as an example, the work of Makito et al., who investigated in 196,303 patients, the association between the type of general anesthesia (propofol-based total intravenous anesthesia versus volatile anesthesia) and cancer progression in patients with gastrointestinal cancers. The authors used instrumental variable analysis to adjust for unmeasured confounding between propofol-based total intravenous anesthesia versus volatile anesthesia patients. The instrumental variable used was total intravenous anesthesia since it reflects the physician’s choice of anesthesia type and is “independent” of unmeasured confounding. While the authors elegantly demonstrated that patients in both anesthesia groups had similar recurrence-free and overall survival, causality could not be determined. Makito used an administrative registry [23]. In such large databases, hospital diagnoses, and procedures are well recorded. However, information on lifestyle or socioeconomic factors that may have influenced cancer recurrence is sparse [23,24].

Well-designed and well-conducted randomized controlled trials are still considered the gold standard in clinical research. Since the early 1980s, anesthesiologists and surgeons have used randomized controlled trials to determine the effectiveness of a new intervention or treatment on perioperative outcomes such as postoperative pain, nausea, vomiting, postoperative complications, and mortality. Clinical investigators use randomization to minimize bias and to determine the cause–effect relationships, which cannot be determined with retrospective studies.

A traditional concept when designing randomized controlled trials was that they should be based on clinical equipoise or the uncertainty principle [25]. Equipoise exists when the investigator or group of investigators has no good basis for choosing between two or more treatment options that may affect a measurable outcome—say, regional versus general anesthesia on cancer recurrence [25]. However, more recently, randomized controlled trials have been designed toward a directional hypothesis—positive expected value—in which investigators try to demonstrate the effectiveness of one intervention over another [26]. As an example, Sessler et al. conducted a randomized controlled trial “to test the primary hypothesis that local or metastatic recurrence after potentially curative breast cancer surgery is reduced in women randomly allocated to regional anesthesia analgesia (paravertebral block and propofol) than in those assigned to general anesthesia with the volatile anesthetic sevoflurane and opioid analgesia.” In Sessler’s study, the positive expected value from the patient’s point of view was a reduction in recurrence if allocated to the regional anesthesia–analgesia group [21]. Another example is a Vijayakumar et al. study, in which naltrexone was compared to placebo to evaluate therapy response in patients with estrogen-positive breast cancer [27]. This study was designed toward a directional hypothesis, i.e., positive expected value, conducted in the nonoperative setting, and naltrexone or placebo was given for up to eight weeks. Although an earlier retrospective study suggested that mutations of the opioid receptor could be implicated in breast cancer progression, Vijayakumar’s study demonstrated minimal effect on tumor response, as measured by positron emission tomography-computed tomography [27,28].

Randomized controlled trials may have issues related to their design, including unclear hypotheses, multiple objectives, poor selection of endpoints, and generalizability problems. Let us consider some of these issues in perioperative oncology (Table 2). Ideally, phase II trials should test interventions’ effect on surrogate outcome markers. In contrast, phase III trials should be focused on “harder” endpoints, such as those measuring quality of life or mortality. Going back to Sessler’s work, the trial (phase III) demonstrated no oncological benefits of regional anesthesia, even though the findings of at least two small randomized controlled trials demonstrated significant improvements with paravertebral analgesia in biomarkers related to cancer progression [21,29,30]. This example highlights the importance of conducting phase III randomized controlled trials and not over-relying on the results of phase II trials.

The generalizability of randomized controlled trials is an important challenge. First, participants who volunteer to participate in randomized controlled trials might not necessarily represent the general population. Second, if exclusion criteria are overly complex, they might reduce the generalizability of the final study results. Although it can be argued that while overly “inclusive” trials might facilitate enrollment and make results more generalizable, they might also “dilute” the treatment effects.

Randomized controlled trials may also present challenges related to conducting them, such as high cost, duration, low recruitment, and follow-up loss [31]. Low recruitment is a frequently encountered problem with randomized controlled studies with inadequate exclusion criteria, significant refusal of consent, or a small number of recruitable patients (rare diseases) [31]. Low recruitment can lead to prolonged study duration. This can be more problematic in studies dealing with long-term endpoints, such as overall survival, or event-driven trials if the rate of the binary outcome is low. Analyzing survival or event-drive data requires specific statistical methods that can deal with censored data or prespecified analysis [12,32]. Surrogate endpoints of overall survival, such as recurrence-free or progression-free survival, are also commonly used to measure the effectiveness of interventions in oncological patients due to a shorter observation period. However, for some malignancies, neither recurrence-free nor progression-free survival necessarily correlates to mortality [33,34].

Loss to follow-up is a significant challenge in studies with long-term primary endpoints such as survival. A ≤5% loss of long-term follow-up is acceptable. For instance, Sessler et al. reported a lower than 5% rate in each arm of their study, indicating good retention of participants up to their last follow-up [21]. While incentives for patients to remain in the study can be used to avoid a loss to follow-up, factors such as changes in geographical location or disappointment with healthcare-related outcomes may discourage participants from staying in the study.

Academic researchers must often rely on public funding to conduct large clinical investigations. In the United States, the main public funding sources are the National Health Institute (NIH) and the Patient-Centered Outcomes Research Institute (PCORI). These institutes have granted significant awards to investigators to conduct large studies such as REGAIN and, more recently, the KALPAS and THRIVE trials. However, neither the NIH nor PCORI are actively funding large clinical trials to determine the effectiveness of different anesthetics or anesthesia techniques on cancer recurrence as primary endpoints.

Outside of the United States, the Public Health Research Institute in Canada, the Australian government’s National Health and Medical Research Council, and the Health Research Council in New Zealand have sponsored important clinical trials such as the POISE-2, POISE-3, and BALANCED trials [35,36,37]. Notably, the Australian government’s National Health and Medical Research Council and the Health Research Council in New Zealand are sponsoring the VAPOR-C trial [38]. This study investigates the effect of propofol-based total venous anesthesia versus volatile anesthesia and intravenous lidocaine versus no-lidocaine in patients undergoing lung and colorectal cancer surgery.

Lastly, important randomized controlled trials have been conducted in China with full or partial public funding support. For instance, Zhang et al. recently reported the findings of a randomized controlled trial evaluating the effect of intravenous lidocaine (bolus followed by an infusion) versus placebo in 563 patients who underwent pancreatic cancer surgery [39]. The study did not demonstrate an oncological benefit associated with the use of lidocaine [39]. Xu et al. conducted a randomized controlled trial to determine the impact of general anesthesia alone plus postoperative intravenous analgesia or combined epidural–general anesthesia plus postoperative epidural analgesia on lung cancer recurrence [40]. The study enrolled 400 patients from 2015 to 2017. In line with Sessler’s study on women undergoing mastectomy, Xu et al. reported that regional anesthesia has no significant impact on lung cancer progression after intended curative surgery [40].

The pharmaceutical industry has significantly increased the financial support for biomedical research in areas such as biologics, especially in rheumatology, neurology, and oncology fields. The main driver of such an increment in support is linked to the market value for biologics, which was approximately 1 trillion dollars in 2016. In contrast, the market value for anesthesia was 7.03 billion dollars in 2021—a substantially smaller market value [41]. Hence, there are small incentives to invest in developing new anesthetics or new technologies in the private sector [42], and this significantly impacts the amount of financial support and investment that pharmaceutical or technology companies provide to investigators in the perioperative care arena. The good news is that the market value for anesthesia and technologies in perioperative medicine will steadily increase by 2030 to 9.56 billion dollars [42]. With an aging population in Northern Europe and the United States, there will be a rise in the incidence of certain cancers and cardiovascular and neurological diseases for which surgery and anesthesia will be integral parts of the care [42]. The challenge for anesthesiologists and researchers will be in establishing partnerships with the industry to generate a high level of evidence.

## 3. Conclusions

The perioperative period is critical for the survival of patients with cancer. The only promising therapies that could influence long-term outcomes in patients with cancer undergoing surgery are a combination of beta-blockers and nonsteroidal anti-inflammatory drugs. Challenges in conducting and interpreting the results of in vitro and in vivo preclinical investigations and multiple retrospective studies have led anesthesiologists to believe that anesthetics strongly impact the mechanisms of cancer progression. Randomized controlled trials also present their challenges. However, only the results of well-designed and well-conducted randomized controlled studies will answer whether the short-term effect of anesthetics or analgesics significantly impacts the survival of patients with cancer. While we wait for the results of those studies, anesthesiologists and researchers should focus their efforts on developing new strategies to improve the quality of life of patients in need of surgical procedures.

## Figures and Tables

**Table 1:** Challenges and limitations of retrospective studies.

Characteristic	Limitations/Challenges
Research or administrative database	Difficult to validate. Created for billing purposes, not for research.
Quality of data	Low quality due to confounding. Relies on the accuracy of written records. Susceptible to selection, memory, and prescription biases. Proper “controls” are difficult to obtain. Not recommended for determining incidence.
Population	Sometimes poorly defined. Definitions of the study disease or outcome may change over time. Not a good source for very rare diseases.
Outcomes	Usually poorly defined and captured due to missing data. Does not allow calculating relative risks.
Interpretation of the results	Cannot determine causality. Demonstrate an association between the exposure and the outcomes. Results are, at best, hypothesis-generating. Not generalizable due to selection bias.

**Table 2:** Challenges and limitations of prospective studies.

Characteristic	Limitations/Challenges
Funding	Typically expensive studies. High cost per patient. Federal funding is usually low.
Patient accrual/Monitoring	Typically low. Even more challenging to investigate infrequent adverse effects. Extensive on-site monitoring.
Clinical research personnel	Complex studies may require personnel with expertise in the perioperative environment.
Outcomes	Usually long-term, recurrence or death. Surrogate outcomes do not always correlate with overall survival. Endpoint adjudication can be difficult. Establishing cancer recurrence or progression is subject to error bias. The definition of “clinically meaningful” can be challenging.
Hypothesis	Equipoise between treatment options versus directional hypothesis testing.
Validity	It may lack generalizability since recruited patients may differ from the population of interest (volunteer bias). Loss of follow-up may threaten the validity of outcomes.
